# Factors associated with fear of childbirth among pregnant adolescents in Northeastern Thailand: A cross-sectional study

**DOI:** 10.18332/ejm/219761

**Published:** 2026-04-30

**Authors:** Umaporn Kuasit, Denlaong Na-sangiem

**Affiliations:** 1 Maternal-Newborn Nursing and Midwifery Department, Faculty of Nursing, Mahasarakham University, Kantharawichai District, Maha Sarakham Province, Thailand

**Keywords:** fear of childbirth, pregnant adolescents, self-efficacy, self-esteem, social support

## Abstract

**INTRODUCTION:**

Fear of childbirth (FOC) is a significant psychological concern among pregnant adolescents, who often experience unplanned pregnancies and limited preparedness for childbirth. This study aimed to examine the level of FOC and identify its psychosocial correlates among pregnant adolescents in Northeastern Thailand.

**METHODS:**

A cross-sectional study was conducted among 147 pregnant adolescents attending antenatal care clinics. Data were collected using validated questionnaires assessing fear of childbirth, anxiety, self-esteem, childbirth self-efficacy, marital relationship, and social support. Descriptive statistics, Pearson’s correlation, and multiple linear regression analyses were performed.

**RESULTS:**

Participants reported a moderate level of FOC (mean=1.62, SD=0.855). Correlation analysis indicated that anxiety was positively associated with FOC, while self-esteem, childbirth self-efficacy, marital relationship, and social support were negatively associated. Multiple linear regression analysis showed that self-esteem (β=0.243, p=0.020) and social support (β= -0.288, p=0.022) were significantly associated with FOC. The model explained 59% of the variance (R^2^=0.59).

**CONCLUSIONS:**

FOC among pregnant adolescents is associated with key psychosocial factors, particularly self-esteem and social support. Strengthening psychological resilience and supportive environments may help reduce childbirth fear and improve maternal outcomes.

## INTRODUCTION

Adolescent pregnancy remains a significant public health concern, particularly in low- and middle-income settings, where young mothers face increased risks of obstetric complications and adverse neonatal outcomes^1,2^. In addition to physical health risks, adolescent pregnancy is often accompanied by psychosocial challenges, including limited education, economic dependence, and insufficient social support^3^.

Fear of childbirth (FOC) is a multidimensional psychological condition characterized by concerns about labor pain, complications, and loss of control^4,5^. Previous studies have demonstrated that FOC is associated with increased anxiety, preference for cesarean delivery, prolonged labor, and adverse maternal outcomes^6-8^. Adolescents may be particularly vulnerable due to a lack of childbirth experience, limited knowledge, and unplanned pregnancies⁸. Psychosocial factors such as self-esteem, self-efficacy, marital relationship, and social support have been identified as important determinants of childbirth fear^9-11^. Higher self-esteem and strong social support are associated with reduced fear, while psychological vulnerability increases fear levels^12,13^.

Despite growing evidence globally, limited studies have specifically examined FOC among pregnant adolescents in Thailand. Therefore, this study aims to examine the level of fear of childbirth and its psychosocial correlates self-esteem, childbirth self-efficacy, marital relationship, and social support among pregnant adolescents in Northeastern Thailand. Our research objectives were to examine the level of fear of childbirth among pregnant adolescents and to investigate the relationships between personal and environmental factors and fear of childbirth among pregnant adolescents.

### Study design and participants

This study employed a cross-sectional design to examine factors associated with fear of childbirth among pregnant adolescents. Data were collected between March and September 2023 from antenatal care clinics in regional and general hospitals in Northeastern Thailand.

Participants were pregnant adolescents aged <20 years (age calculated at the expected date of delivery). The required sample size was calculated using G*Power software for multiple linear regression analysis (effect size f^2^=0.15, α=0.05, power=0.90), resulting in a minimum sample size of 147 participants.

Participants were eligible if they were able to communicate in Thai, had no severe physical or psychiatric conditions, and were willing to participate. Adolescents with serious medical or mental health conditions that could interfere with participation were excluded.

Eligible participants were approached during antenatal care visits by trained research assistants. The purpose of the study was explained, and written informed consent was obtained prior to participation. Participants completed self-administered questionnaires in a private setting, which took approximately 25–30 minutes.

### Ethical considerations

This study was approved by the Institutional Review Board (IRB) of Mahasarakham University, Thailand (Reference No. 337-314/2565). All participants were fully informed about the study objectives, procedures, confidentiality, and their right to withdraw at any time. Written informed consent was obtained from all participants prior to data collection. For participants aged <20 years, assent was obtained from the participants and informed consent from their parents/guardians. Confidentiality and anonymity were strictly maintained throughout the study.

### Measures

Data were collected using structured questionnaires consisting of demographic characteristics and validated instruments measuring fear of childbirth and psychosocial factors^14,15^.

Fear of childbirth was assessed using a 16-item scale adapted from Fisher et al. and modified for Thai populations. Each item was rated on a 5-point Likert scale ranging from 0 (not at all) to 4 (extremely), with a total score ranging from 0 to 64. Higher scores indicate greater fear of childbirth. The scale demonstrated high internal consistency (Cronbach’s α=0.91).

Self-esteem was measured using the Rosenberg Self-Esteem Scale, consisting of 10 items rated on a 4-point Likert scale. Higher scores indicate higher levels of self-esteem. The reliability coefficient in this study was acceptable (Cronbach’s α=0.76).

Childbirth self-efficacy was measured using the Childbirth Self-Efficacy Inventory developed by Lowe. The instrument consists of 16 items rated on a 10-point scale, reflecting confidence in coping with labor. Higher scores indicate greater self-efficacy. The scale demonstrated excellent reliability (Cronbach’s α=0.96).

Marital relationship was assessed using the Dyadic Adjustment Scale, which includes 28 items covering relationship satisfaction, cohesion, consensus, and affection. Higher scores indicate better relationship quality. The reliability coefficient was 0.82.

Social support was measured using a questionnaire based on House’s social support theory, consisting of 10 items rated on a 5-point Likert scale. Higher scores indicate greater perceived social support. The scale demonstrated good reliability (Cronbach’s α=0.80).

### Data analysis

Data was analyzed using SPSS software. Descriptive statistics, including frequency, percentage, mean, and standard deviation, were used to describe participants’ characteristics and study variables. Pearson’s correlation coefficient was used to examine the relationships among fear of childbirth and psychosocial variables. Multiple linear regression analysis was performed to identify factors associated with fear of childbirth. Independent variables entered into the model included self-esteem, childbirth self-efficacy, marital relationship, and social support. All statistical tests were two-tailed, and statistical significance was set at p<0.05.

## RESULTS

### Participant characteristics

A total of 147 pregnant adolescents were included in this study. The majority were aged 15–18 years (56.5%), followed by those aged >18 years (37.3%). Most participants had completed lower or upper secondary education (67.5%), and a substantial proportion were unemployed (45.8%) or students (30.8%). Most participants were primigravidas (86.6%) and had no history of childbirth (90.0%). More than half of the pregnancies were unplanned (53.3%). The majority initiated antenatal care before 12 weeks of gestation (60.5%) and reported no chronic conditions (84.2%). Regarding social context, most participants lived with their partners and family members, and 71.7% identified their partners as the primary caregivers during pregnancy (Table 1).

**Table 1:** Descriptive characteristics of pregnant adolescents, cross-sectional study conducted in Northeastern Thailand, 2022 (N=147)

*Characteristics*	*n (%)*
**Participant age** (years)	
<15	5 (4.2)
15–18	69 (56.5)
>18	46 (37.3)
**Partner age** (years)	
<20	43 (36.1)
20–25	49 (56.5)
>25	46 (37.3)
**Participant education level**	
Primary school	11 (9.2)
Lower secondary	42 (35.0)
Upper secondary	39 (32.5)
Vocational	24 (20.0)
Bachelor’s degree or higher	4(3.3)
**Partner education level**	
Primary school	10 (6.5)
Lower secondary	45 (37.5)
Upper secondary	37 (30.8)
Vocational	24 (20.0)
Bachelor’s degree or higher	1 (0.8)
Other	4(3.3)
**Marital status**	
Single	28 (23.3)
Living together	74 (61.7)
Separated	1 (0.8)
Other	17 (14.2)
**Participant occupation**	
Student	37 (30.8)
Unemployed	55 (45.8)
Labor	12 (10.0)
Merchant	8 (6.7)
Agriculture	4(3.3)
Other	4(3.3)
**Partner occupation**	
Labor	69 (57.5)
Student	13 (10.8)
Merchant	9 (7.5)
Agriculture	8 (6.7)
Government officer/state enterprise	1 (0.8)
Unemployed	19 (15.8)
**Religion**	
Buddhist	118 (98.3)
Muslim	2 (1.7)
**Living arrangement**	
With partner only	36 (30.0)
With partner and own parents	28 (23.3)
With partner and partner’s parents	34 (28.3)
With own parents	21 (17.5)
Alone	1 (0.8)
**Primary caregiver during pregnancy**	
Partner	86 (71.7)
Own parents	21 (17.5)
Partner’s parents	9 (7.5)
Friends	2 (1.7)
None	4 (3.3)
**Gravidity**	
First pregnancy	104 (86.6)
Second pregnancy	16 (13.3)
**History of abortion**	
No	114 (95.0)
Yes	6 (5.0)
**History of delivery**	
No	108 (90.0)
Yes	12 (10.0)
Vaginal delivery	10 (8.4)
Cesarean section	4 (3.3)
**Gestational age** (weeks)	
<12	30 (26.1)
12–28	50 (44.3)
>28	20 (29.6)
**First antenatal care visit**	
<12 weeks	69 (60.5)
≥12 weeks	45 (39.4)
**Planned pregnancy**	
Yes	55 (45.8)
No	64 (53.3)
**Chronic conditions during pregnancy**	
None	101 (84.2)
Yes	19 (15.8)
Diabetes	18 (15.0)
Hypertension	1 (0.8)
Heart disease	2 (1.6)
Thyroid disease	1 (0.8)
Anemia/thalassemia	11 (9.2)
Syphilis	4 (3.3)
**Pregnancy complications**	
None	101 (84.2)
Yes	18 (15.0)

### Levels of fear of childbirth and psychosocial variables

Participants reported a moderate level of fear of childbirth (mean=1.62, SD=0.86). Self-esteem was at a moderate level (mean=2.67, SD=0.42), while childbirth self-efficacy (mean=6.32, SD=2.35) and marital relationship (mean=2.82, SD=0.67) were high. Social support was reported at a very high level (mean=3.66, SD=0.87). Pearson’s correlation analysis showed that psychosocial variables were associated with fear of childbirth. Correlation coefficients ranged from 0.016 to 0.649, indicating low to moderate relationships among variables (Table 2).

**Table 2:** Correlation matrix among fear of childbirth and psychosocial variables, cross-sectional study conducted in Northeastern Thailand, 2022 (N=147)

*Variables*	*1*	*2*	*3*	*4*	*5*
**1. Fear of childbirth**	1.00				
**2. Self-esteem**	0.016	1.00			
**3. Childbirth self-efficacy**	0.314	0.116	1.00		
**4. Marital relationship**	0.250	0.478	0.449	1.00	
**5. Social support**	0.154	0.340	0.493	0.649	1.00

Pearson’s correlation coefficients.

### Factors associated with fear of childbirth

Multiple linear regression analysis was performed to examine factors associated with fear of childbirth among pregnant adolescents. The overall model was statistically significant (F=2.665, p=0.036) and explained 59% of the variance in fear of childbirth (R^2^=0.59). Among the variables included in the model, self-esteem (β=0.243; 95% CI: 0.083–0.919, p=0.020) and social support (β= -0.288; 95% CI: -0.523–0.043, p=0.022) were significantly associated with fear of childbirth. Childbirth self-efficacy (β=0.124; 95% CI: -0.032–0.122, p=0.246) and marital relationship (β=0.078; 95% CI: -0.228–0.428, p=0.550) were not statistically significant.

The positive standardized coefficient for self-esteem suggests that this variable contributes to variation in fear of childbirth within the model, although the direction of the association should be interpreted with caution. In contrast, social support showed a negative association, indicating that higher levels of perceived support were related to lower levels of childbirth fear. Overall, these findings indicate that psychosocial factors, particularly self-esteem and social support, play an important role in explaining differences in fear of childbirth among pregnant adolescents. The model explained 59% of the variance in fear of childbirth (R^2^=0.59) (Table 3).

**Table 3:** Multiple linear regression analysis of psychosocial factors associated with fear of childbirth among pregnant adolescents, cross-sectional study, Northeastern Thailand (Ν=147)

*Variables*	*B*	*SE*	*β*	*95% CI for B*	*p*
Constant	0.757	0.530	-	-0.289–1.803	0.156
Self-esteem	0.501	0.212	0.243	0.083–0.919	0.020*
Childbirth self-efficacy	0.045	0.039	0.124	-0.032–0.122	0.246
Marital relationship	0.100	0.166	0.078	-0.228–0.428	0.550
Social support	-0.283	0.122	-0.288	-0.523–0.043	0.022*
Model statistics: R^2^=0.59					

Multiple linear regression analysis including self-esteem, childbirth self-efficacy, marital relationship, and social support. SE: standard error.

* p<0.05.

## DISCUSSION

This study examined psychosocial factors associated with fear of childbirth (FOC) among pregnant adolescents and found that overall FOC was at a moderate level. This finding is consistent with previous studies indicating that adolescent mothers often experience moderate to high levels of childbirth fear due to limited childbirth experience, uncertainty, and psychological vulnerability^14,16-18^.

Self-esteem and social support were identified as significant factors associated with FOC. Adolescents with higher self-esteem tended to report lower levels of childbirth fear, suggesting that internal psychological resources play an important role in shaping emotional responses to childbirth^19,20^. This finding is consistent with previous research demonstrating that women with higher self-esteem and self-efficacy perceive childbirth as more manageable and less threatening^15,21,22^.

Similarly, social support was significantly associated with lower levels of childbirth fear. Adolescents who received emotional and informational support from partners, family members, and healthcare providers experienced reduced fear^23-25^. This finding is consistent with earlier studies showing that strong social support networks can buffer stress and reduce anxiety during pregnancy, particularly among vulnerable populations such as adolescents^26,27^. In contrast, childbirth self-efficacy and marital relationships were not statistically significant in the regression model, although they were correlated with FOC. This suggests that these factors may have indirect effects or may be mediated by broader psychosocial constructs, such as self-esteem and perceived social support^28-30.^

The findings of this study can be interpreted within the framework of Bandura’s Social Cognitive Theory^31^, which emphasizes the interaction between personal and environmental factors in shaping behavior and emotional responses^32,33^. In this context, self-esteem represents an internal psychological factor, whereas social support reflects external environmental influences. The interaction between these factors may influence how adolescents perceive and cope with childbirth-related stress^34^. These findings highlight the importance of psychosocial determinants in understanding fear of childbirth among adolescents. Strengthening internal psychological resources, such as self-esteem, together with enhancing social systems, as indicated by our results, may help reduce childbirth-related fear and improve maternal psychological well-being during pregnancy within our setting.

### Strengths and limitations

This study has several strengths, including the use of validated instruments and the focus on a specific vulnerable population. However, several limitations should be considered. First, the cross-sectional design limits causal interpretation. Second, purposive sampling may affect the generalizability of the findings. Third, self-reported data may be subject to response bias.

## CONCLUSIONS

Fear of childbirth among pregnant adolescents in Thailand is associated with key psychosocial factors, particularly self-esteem and social support. Strengthening these factors may contribute to improved maternal psychological well-being and childbirth experiences within the Thai setting.

## Data Availability

The data supporting this research are available from the authors on reasonable request.
